# Inhibition of renin–angiotensin system affects prognosis of advanced pancreatic cancer receiving gemcitabine

**DOI:** 10.1038/sj.bjc.6605955

**Published:** 2010-10-26

**Authors:** Y Nakai, H Isayama, H Ijichi, T Sasaki, N Sasahira, K Hirano, H Kogure, K Kawakubo, H Yagioka, Y Yashima, S Mizuno, K Yamamoto, T Arizumi, O Togawa, S Matsubara, T Tsujino, K Tateishi, M Tada, M Omata, K Koike

**Affiliations:** 1 Department of Gastroenterology, Graduate School of Medicine, The University of Tokyo, 7-3-1 Hongo Bunkyo-ku, Tokyo 113-8655, Japan; 2 Yamanashi Prefectural Central Hospital Organization, 1-1-1 Fujimi, Kofu, Yamanashi 400-8506, Japan

**Keywords:** angiotensin I-converting enzyme inhibitors, angiotensin II type-1 receptor blockers, chemotherapy, gemcitabine, pancreatic cancer, renin–angiotensin system

## Abstract

**Background:**

The renin–angiotensin system (RAS) is thought to have a role in carcinogenesis, and RAS inhibition may prevent tumour growth.

**Methods:**

We retrospectively investigated the impact of angiotensin I-converting enzyme inhibitors (ACEIs) and angiotensin II type-1 receptor blockers (ARBs) in 155 patients with pancreatic cancer receiving gemcitabine monotherapy. Patients were divided into three groups: the ACEI/ARB group (27 patients receiving an ACEI or ARB for hypertension (HT)), the non-ACEI/ARB with HT group (25 patients receiving antihypertensive drugs other than ACEIs or ARBs), and the non-HT group (103 patients receiving no antihypertensive drugs).

**Results:**

Patient characteristics were not different, except for age and HT medications. Progression-free survival (PFS) was 8.7 months in the ACEI/ARB group, 4.5 months in the non-ACEI/ARB with HT group, and 3.6 months in the non-HT group. Overall survival (OS) was 15.1 months in the ACEI/ARB group, 8.9 months in the non-ACEI/ARB with HT group, and 9.5 months in the non-HT group. The use of ACEIs/ARBs was a significant prognostic factor for both PFS (*P*=0.032) and OS (*P*=0.014) in the multivariate analysis.

**Conclusions:**

The ACEIs/ARBs in combination with gemcitabine might improve clinical outcomes in patients with advanced pancreatic cancer. Prospective trials are needed to test this hypothesis.

Systemic administration of gemcitabine has been the standard chemotherapy for advanced pancreatic cancer since Burris *et al* (1997) demonstrated the superiority of gemcitabine over 5-flurouracil. Combination therapies of gemcitabine with other cytotoxic drugs (Berlin *et al*, 2002; Louvet *et al*, 2005; Herrmann *et al*, 2007; Cunningham *et al*, 2009; Nakai *et al*, 2009, 2010) have been thoroughly investigated, but only two randomised control trials have shown significant improvements in the survival so far (Reni *et al*, 2005; Conroy *et al*, 2010). Many molecular target drugs have been recently investigated in clinical trials (Van Cutsem *et al*, 2009; Kindler *et al*, 2010; Philip *et al*, 2010). Erlotinib in combination with gemcitabine was the only drug that showed prolonged survival in advanced pancreatic cancer (Moore *et al*, 2007) but the survival benefit was modest, with only a 2-week improvement in survival, and was accompanied by high costs and greater toxicity than gemcitabine alone. Thus, more effective and safe drugs are awaited.

The systemic renin–angiotensin system (RAS) is associated with cardiovascular regulation and angiotensin I-converting enzyme inhibitors (ACEIs) and angiotensin II type-1 receptor blockers (ARBs) are some of the most widely used antihypertensive drugs. Since Lever *et al* (1998) reported that the use of ACEI was associated with a decreased incidence of cancer in a large cohort study, the potential role of the local RAS in carcinogenesis has attracted substantial attention. The local RAS reportedly promotes angiogenesis and proliferation via vascular endothelial growth factor (VEGF) expression or epidermal growth factor receptor (EGFR) expression (Ager *et al*, 2008; Khakoo *et al*, 2008). Synergistic inhibition of tumour growth in a murine pancreatic cancer has been demonstrated with combined gemcitabine and losartan treatment via VEGF suppression (Noguchi *et al*, 2009). In addition, the inhibition of RAS is also reported to induce apoptosis in pancreatic cancer cells (Amaya *et al*, 2004; Gong *et al*, 2010). Thus, the use of ACEIs or ARBs may inhibit tumour growth in patients with pancreatic cancer. In this study, we retrospectively analysed clinical outcomes in patients with pancreatic cancer receiving gemcitabine monotherapy to clarify the impact of ACEIs and ARBs.

## Patients and methods

### Patients

All patients with locally advanced and metastatic pancreatic cancer who received first-line chemotherapy with gemcitabine monotherapy without previous treatment, including surgical resection and radiotherapy at the University of Tokyo Hospital between April 2001 and August 2009 were retrospectively studied. The use of hypertension (HT) medications including ACEIs or ARBs was retrospectively retrieved from the medical records, and patients were divided into three groups: the ACEI/ARB group (patients who received ACEIs or ARBs for HT), the non-ACEI/ARB with HT group (patients who received antihypertensive drugs other than ACEIs or ARBs), and the non-HT group (patients who did not receive antihypertensive drugs). This study was approved by The University of Tokyo Hospital ethics committee.

### Treatment and tumour response

Gemcitabine was administered at a dose of 1000 mg m^−2^ in a 30-min intravenous infusion on days 1, 8, and 15 in 4-week cycles. The relative dose intensity (RDI) for gemcitabine was defined as the ratio of the actual dose intensity to the standard dose intensity. Tumour response was assessed via computed tomography using the Response Evaluation Criteria in Solid Tumours version 1.0 (Therasse *et al*, 2000). The evaluation was repeated every two courses, or more frequently in patients with clinically suspected progression.

### Statistical methods

Overall survival (OS) and progression-free survival (PFS) were estimated using the Kaplan–Meier method and compared using the log-rank test. The *χ*^2^-test or Fisher’s exact test was used to compare categorical variables. The independent *t*-test, Mann–Whitney *U-*test, or Kruskal–Wallis test was used to compare continuous variables as appropriate. All reported *P*-values were the result of two-sided tests, with *P*<0.05 considered statistically significant.

To exclude possible confounding factors, the Cox proportional hazards model was used to estimate hazard ratios of the use of ACEIs/ARBs adjusted for significant prognostic factors. Prognostic factors included age (<65 or ⩾65 years old), gender (male or female), performance status (PS; 0–1 or ⩾2), distant metastasis (yes or no), pretreatment carbohydrate antigen 19-9 level, and treatment group (the ACEI/ARB group, the non-ACEI/ARB with HT group, or the non-HT group). Prognostic factors with *P*<0.05 in the univariate analysis were included in the multivariate analysis.

## Results

### Patients’ characteristics

In total, 155 patients received first-line gemcitabine monotherapy between April 2001 and August 2009 at The University of Tokyo Hospital, with a median follow-up time of 9.5 months. In all, 52 patients received medication for HT (Table 1) and of these, 27 patients took an ACEI (*n*=6) or ARB (*n*=21). Other antihypertensive drugs included calcium-channel blockers (*n*=22) and *β*-blockers (*n*=3). The most commonly administered drug was candesartan (*n*=12). The doses of ACEIs and ARBs were as follows: enalapril 5 mg in three patients and 2.5 mg in one patient, lisinopril 10 mg in one patient, temocapril 4 mg in one patient, candesartan 4 mg in eight patients and 8 mg in four patients, losartan 25 mg in four patients, olmesartan 10 mg in three patients and valsartan 40 mg in two patients. Except one patient in the ACEI/ARB group, all the patients with HT continued to receive their antihypertensive drugs at least during their chemotherapy. One patient in the ACEI/ARB group stopped taking valsartan 1 month after starting chemotherapy because of the decrease in blood pressure. Patient characteristics of the ACEI/ARB group (*n*=27), the non-ACEI/ARB with HT group (*n*=25), and the non-HT group (*n*=103) are shown in Table 2. Baseline characteristics did not differ significantly among groups, apart from age and HT medications. The mean RDI of gemcitabine was 67.3% in the ACEI/ARB group, 64.6% in the non-ACEI/ARB with HT group, and 66.4% in the non-HT group (*P*=0.914). At the time of analysis, five patients in the ACEI/ARB group and two patients each in the non-ACEI/ARB with HT and non-HT groups continued to receive gemcitabine without disease progression, with a median follow-up time of 7.9 months (range, 5.2–17.3 months). Among 146 patients who showed disease progression during gemcitabine treatment, second-line chemotherapy was administered in 23.8% of the ACEI/ARB group, 52.2% of the non-ACEI/ARB with HT group, and 33.7% of the non-HT group (*P*=0.134). Four patents (2.6%) were lost to follow after disease progression; one patient in the ACEI/ARB group, one patient in the non-ACEI/ARB with HT group, and two patients in the non-HT Group. The median follow-up period of these four patients was of 7.7 months.

### Impact of ACEIs/ARBs on clinical outcomes

**Figure 1:**
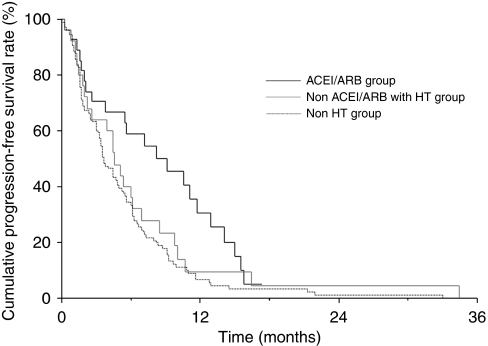
Kaplan–Meier curves for progression-free survival by treatment groups. The median progression-free survival was 8.7 months in the ACEI/ARB group, 4.5 months in the non-ACEI/ARB with HT group, and 3.6 months in the non-HT group.

**Figure 2:**
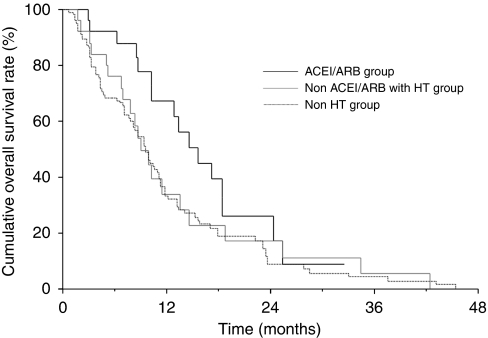
Kaplan–Meier curves for overall survival by treatment groups. The median overall survival was 15.1 months in the ACEI/ARB group, 8.9 months in the non-ACEI/ARB with HT group, and 9.5 months in the non-HT group.

Response rates were comparable among the three groups; 3.7% in the ACEI/ARB group, 4.0% in the non-ACEI/ARB with HT group, and 2.9% in the non-HT group (*P*=0.485), whereas the disease control rate was 63.0% in the ACEI/ARB group compared with 36.0% in the non-ACEI/ARB with HT group and 44.7% in the non-HT group (*P*=0.131). The median PFS (Figure 1) was 8.7 months (95% confidence interval (CI), 2.6–11.1) in the ACEI/ARB group, 4.5 months (95% CI, 2.2–6.1) in the non-ACEI/ARB with HT group, and 3.6 months (95% CI, 3.1–4.8) in the non-HT group (*P*=0.015 by log-rank test). The median OS (Figure 2) was 15.1 months (95% CI, 10.2–18.5) in the ACEI/ARB group, 8.9 months (95% CI, 6.7–11.4) in the non-ACEI/ARB with HT group, and 9.5 months (95% CI, 7.8–11.2) in the non-HT group (*P*=0.140 by log-rank test). There were no significant differences between patients taking ACEIs and ARBs. The median PFS was 10.6 months (95% CI, 1.5–15.1) in patients taking ACEIs and 8.2 months (95% CI, 2.0–12.9) in patients taking ARBs (*P*=0.756 by log-rank test). The median OS was 13.3 months (95% CI, 3.0–24.6) in patients taking ACEIs and 15.6 months (95% CI, 8.7–25.4) in patients taking ARBs (*P*=0.794 by log-rank test).

Although patient characteristics of the three groups were similar among groups except for age and HT medications, we performed Cox proportional hazard analyses to exclude the possible influence of confounding prognostic factors. The Cox univariate and multivariate analyses for PFS and OS are shown in Tables 3 and 4, respectively. The use of ACEIs/ARBs remained significant as a prognostic factor for both PFS and OS, in addition to the previously reported prognostic factors, PS and disease stage. The hazard ratios for the ACEI/ARB group against the non-HT group were 0.58 (*P*=0.032) for PFS and 0.52 (*P*=0.014) for OS. Those for the non-ACEI/ARB group were 0.97(*P*=0.890) for PFS and 1.23 (*P*=0.430) for OS.

## Discussion

This retrospective study is the first report to clarify the clinical impact of the use of ACEIs or ARBs in pancreatic cancer. The use of ACEIs or ARBs was associated with longer PFS and OS in patients with advanced pancreatic cancer receiving gemcitabine monotherapy. These data suggest that inhibition of the RAS in human pancreatic cancer may inhibit tumour growth and improve survival, in accordance with previous *in vitro* studies and *in vivo* animal studies.

ACEIs and ARBs are widely used as antihypertensive drugs, and the reports of organ protective effects (Grandi and Maresca, 2006) by ACEIs are increasing, including inhibition of cardiac hypertrophy, diabetic nephropathy, and diabetic retinopathy. With respect to anticancer effects, Lever *et al* (1998) reported that the long-term use of ACEIs reduced the incidence of cancer in a prospective cohort study, though they did not explore the underlying mechanisms. Since then, in addition to cardiovascular homostasis by the systemic RAS, increasing evidence indicates a role of the local RAS in various aspects of carcinogenesis, including angiogenesis, cell proliferation, apoptosis, and inflammation (Ager *et al*, 2008; Khakoo *et al*, 2008). On the other hand, a meta-analysis denied the reduced cancer incidence with ACEIs (Coleman *et al*, 2008) and the increased risk of cancer incidence was also reported with ARBs (Sipahi *et al*, 2010). Both the clinical impact of inhibition of RAS on cancer incidence and its underlying mechanism remains unclear.

The existence of the local RAS was first reported in the canine pancreas in 1991 (Chappell *et al*, 1991) and in the human pancreas in 1999 (Tahmasebi *et al*, 1999). The local pancreatic RAS has been implicated in various physiological conditions including pancreatitis, fibrosis, and diabetes mellitus (Leung, 2007). The involvement of the local RAS in pancreatic cancer was suggested because of the expression of angiotensin II (Ohta *et al*, 2003) and the angiotensin II type-1 receptor (Fujimoto *et al*, 2001) in human pancreatic cancer. The ACEIs and ARBs inhibit pancreatic cancer cell proliferation *in vitro* (Arafat *et al*, 2007) and also slow murine pancreatic cancer progression *in vivo* via down-regulation of VEGF expression (Noguchi *et al*, 2009; Fendrich *et al*, 2010). Inhibition of RAS is also reported to induce apoptosis in pancreatic cancer cells (Amaya *et al*, 2004; Gong *et al*, 2010). Accordingly, these drugs were suggested to be potential treatments for pancreatic cancer or for the prevention of pancreatic cancer. However, the clinical impact of ACEIs and ARBs in pancreatic cancer treatment has not been fully clarified. With respect to other cancer types, a pilot study reported that ARBs had cytostatic activity in hormone-refractory prostate cancer, as indicated by decreased prostate-specific antigen levels (Uemura *et al*, 2005), and the addition of ACEIs/ARBs to platinum-based chemotherapy was associated with prolonged survival in patients with advanced non-small cell lung cancer in a retrospective study (Wilop *et al*, 2009). ACEIs in combination with vitamin K were also reported to suppress the recurrence of hepatocellular carcinoma in a prospective study (Yoshiji *et al*, 2009).

It is possible that ACEIs and ARBs have different influences on cancer because ACEIs block both angiotensin II type-1 and type-2 receptors, whereas ARBs block only type-1 receptor. The role of angiotensin II type-2 receptor is less investigated than angiotensin II type-1 receptor, which is shown to induce angiogenesis, proliferation, and inflammation. Angiotensin II type-2 receptor is reported to be both anti- and pro-angiogenetic (Ager *et al*, 2008). In this study, there were no significant differences in survival between patients taking ACEIs and ARBs. Our study population was too small to analyze the differences between these two types of drugs.

The disappointing results of combination therapy with gemcitabine and cytotoxic drugs have led to intense investigation of molecular target drugs for pancreatic cancer (Burris and Rocha-Lima, 2008). Inhibition of VEGF or EGFR failed to demonstrate significant survival prolongation except one trial with erlotinib (Moore *et al*, 2007). The inhibition of RAS by ACEI or ARB reportedly influences multiple pathways including angiogenesis, proliferation, and apoptosis, and can be a safe and inexpensive strategy against pancreatic cancer, but a prospective study is warranted to evaluate antitumour effects by the inhibition of RAS.

This study had some limitations. As this was a retrospective study in a single institution and the sample size of the ACEI/ARB group was small, unknown sources of bias may exist in the findings. However, other than age and HT medications, no significant differences were detected in patient characteristics among groups, and the multivariate analysis revealed that ACEI/ARB use remained a significant prognostic factor for both PFS and OS, though we cannot fully correct the bias that patients with HT were much older than patients without HT. Gemcitabine dose intensity and the induction rate of second-line chemotherapy were also similar in the three groups. The results of the non-ACEI/ARB with HT group also excluded the possibility that patients who did not receive antihypertensive drugs had a poorer prognosis. However, a prospective study with a larger population is warranted to confirm our hypothesis.

In conclusion, our retrospective analysis suggests that ACEIs or ARBs in combination with gemcitabine may improve clinical outcomes in patients with advanced pancreatic cancer. We have started a phase I trial of candesartan in combination with gemcitabine, which is currently ongoing (UMIN registration number 000002152).

## Figures and Tables

**Table 1:** Number of patients receiving antihypertensive drugs

**Drugs**	**Number of patients**
*ACEI*	6
Enalapril	4
Lisinopril	1
Temocapril	1
*ARB*	21
Candesartan	12
Losartan	4
Olmesartan	3
Valsartan	2
*Calcium-channel blockers*	22
Amlodipine	8
Nifedipine	6
Manidipine	4
Diltiazem	4
*β-Blockers*	3
Atenolol	2
Betaxolol	1

Abbreviations: ACEI=angiotensin I-converting enzyme inhibitor; ARB=angiotensin II type-1 receptor blocker.

**Table 2:** Patient characteristics

**Characteristics**	**ACEI/ARB (*n*=27)**	**Non-ACEI/ARB with HT (*n=*25)**	**Non-HT (*n=*103)**	***P*-value**
Median age, years (range)	71 (53–87)	73 (56–88)	63 (41–89)	<0.001
Gender (male/female)	15/12	11/14	58/45	0.538
*PS*				0.621
0	14	11	40	
1	9	13	47	
2	4	1	14	
3	0	0	2	
*Location*				0.355
Head	12	16	53	
Body/tail	15	9	50	
*Stage*				0.668
Locally advanced	12	10	40	
Metastatic	15	15	63	
*Site of metastasis, n (%)*				
Liver	10 (37.0%)	12 (48.0%)	46 (44.7%)	0.942
Lung	1 (3.7%)	3 (12.0%)	13 (12.6%)	0.473
Lymph node	13 (48.2%)	12 (48.0%)	50 (48.5%)	1.000
Peritoneum	3 (11.1%)	1 (4.0%)	15 (14.6%)	0.412
Median CEA, ng ml^−1^ (range)	4.0 (0.8–120.2)	6.1 (2.4–2964.3)	5.7 (1–2756.9)	0.201
Median CA19-9, U ml^−1^ (range)	490 (1–145600)	421 (1–102100)	324 (1–182600)	0.788
Hypertension	27	25	0	<0.001

Abbreviations: ACEI=angiotensin I-converting enzyme inhibitor; ARB=angiotensin II type-1 receptor blocker; CA19-9=carbohydrate antigen 19-9; CEA=carcinoembryonic antigen; HT=hypertension; PS=performance status.

**Table 3:** Univariate and multivariate analyses for progression-free survival

	**Univariate analysis**		**Multivariate analysis**	
**Factor**	**HR (95% CI)**	***P*-value**	**HR (95% CI)**	***P*-value**
*Age, years*				
<65	1	0.032	1	0.605
⩾65	0.69 (0.50–0.97)		0.90 (0.61–1.33)	
*Gender*				
Male	1	0.387		
Female	0.87 (0.62–1.20)			
*PS*				
0–1	1	<0.001	1	0.026
⩾2	2.69 (1.64–4.21)		2.04 (1.09–3.58)	
*Stage*				
Locally advanced	1	0.002	1	0.031
Metastatic	1.70 (1.21–2.40)		1.47 (1.04–2.10)	
*CA19-9*				
Per 1000 increase	1.01 (1.00–1.01)	0.023	1.00 (0.99–1.01)	0.559
*Group*				
Non-HT	1		1	
Non-ACEI/ARB with HT	0.79 (0.49–1.22)	0.294	0.97 (0.58–1.56)	0.890
ACEI/ARB	0.51 (0.31–0.80)	0.003	0.58 (0.34–0.95)	0.032

Abbreviations: ACEI=angiotensin I-converting enzyme inhibitor; ARB=angiotensin II type-1 receptor blocker; CA19-9=carbohydrate antigen 19-9; CI=confidence interval; HR=hazard ratio; HT=hypertension; PS=performance status.

**Table 4:** Univariate and multivariate analyses for overall survival

	**Univariate analysis**		**Multivariate analysis**	
**Factor**	**HR (95% CI)**	***P*-value**	**HR (95% CI)**	***P*-value**
*Age, years*				
<65	1	0.142		
⩾65	0.76 (0.53–1.10)			
*Gender*				
Male	1	0.041	1	0.006
Female	0.69 (0.48–0.98)		0.59 (0.40–0.86)	
*PS*				
0–1	1	<0.001	1	<0.001
⩾2	4.14 (2.42–6.76)		4.08 (2.22–7.05)	
*Stage*				
Locally advanced	1	0.007	1	0.030
Metastatic	1.65 (1.14–2.41)		1.69 (1.16–2.47)	
*CA19-9*				
Per 1000 increase	1.01 (1.01–1.02)	0.001	1.01 (1.00–1.01)	0.058
*Group*				
Non-HT	1		1	
Non-ACEI/ARB with HT	0.92 (0.55–1.45)	0.718	1.23 (0.73–1.98)	0.430
ACEI/ARB	0.59 (0.33–0.97)	0.038	0.52 (0.29–0.88)	0.014

Abbreviations: ACEI=angiotensin I-converting enzyme inhibitor; ARB=angiotensin II type-1 receptor blocker; CA19-9=carbohydrate antigen 19-9; CI=confidence interval; HR=hazard ratio; HT=hypertension; PS=performance status.
